# Iodixanol as a Contrast Agent in a Fibrin Hydrogel for Endodontic Applications

**DOI:** 10.3389/fphys.2017.00152

**Published:** 2017-03-15

**Authors:** Gabriel Hertig, Matthias Zehnder, Anna Woloszyk, Thimios A. Mitsiadis, Anja Ivica, Franz E. Weber

**Affiliations:** ^1^Oral Biotechnology and Bioengineering, University of ZurichZurich, Switzerland; ^2^Preventive Dentistry, Periodontology, and Cariology, University of ZurichZurich, Switzerland; ^3^Orofacial Development and Regeneration, Institute of Oral Biology, University of ZurichZurich, Switzerland

**Keywords:** fibrin gel, regenerative endodontics, iodixanol, contrast agent, tooth

## Abstract

The application of biomaterials used in regenerative endodontics should be traceable. In this study, we checked some basic effects of rendering a fibrin hydrogel radiopaque using an iodine-based contrast agent (iodixanol) approved for systemic application. Fibrin hydrogels were prepared from a fibrin sealant (Tisseel) using either an isotonic iodixanol solution (Visipaque 320, test) or Tris buffer (control) as a diluent. Gelation kinetics, radiopacity, and swelling of lyophilized hydrogels were tested using standard methods. Hydrogel structure was evaluated using scanning electron microscopy (SEM). Furthermore, iodixanol release from the test gels was assessed using spectrophotometry, and tissue compatibility was compared between test and control hydrogels using the chick chorioallantoic membrane (CAM) assay. Results were compared using pairwise *t*-test, *p* < 0.05. Iodixanol caused a 70-fold delay in gelation to 26 min in the test compared to the control hydrogels (22 ± 1 s). Radiopacity of the test gels was 1.9 ± 0.2 mm Al/mm, compared to zero in the control hydrogels. Lyophilized hydrogel swelling was strongly reduced when iodixanol was added to the hydrogel (*p* < 0.05). Test hydrogels had an altered SEM appearance compared to controls, and exhibited a reduced porosity. Iodixanol release from the test hydrogels reached 14.5 ± 0.5% after 120 h and then ceased. This release did not have any apparent toxic effect and neither affected the viability, nor the physiology or vascularization of the CAM of fertilized chicken eggs. Iodixanol can render a fibrin hydrogel radiopaque and maintains its tissue compatibility, yet impacts gelation kinetics and hydrogel porosity.

## Introduction

An exciting new field has emerged in endodontic research over the recent years: Regenerative Endodontics (Hargreaves, 2016). While attempts to attract soft tissues into the necrotic root canal space are not necessarily new (Nygaard Ostby, 1963), the systematic approach in the context of current tissue engineering concepts surely is. Different paths are followed that vary from pure basic science to translational medicine with the development and improvement of clinically applicable protocols. However, the passage to the clinics necessitates specific precautions for tissue engineering concepts and related products (Mao et al., 2012). These include issues such as treatment costs, safety, and also the regulation of medical devices by local administrative bodies.

Currently, in the context of pulp tissue engineering, a so-called cell-free or cell-homing approach is seen as favorable (Kim et al., 2010; Lee et al., 2010). This approach involves the conditioning of the root canal wall by EDTA to release molecular cues for the attraction and differentiation of invading pluripotent cells, followed by the application of a scaffold in the pulp space (Galler, 2016). In the current clinical protocol, this scaffold is a blood clot, which is generated by controlled bleeding through the apical foramen (Trope, 2010). Clinical outcomes with this technique vary (Chen et al., 2012), and negative reports have curbed the initial enthusiasm (Nosrat et al., 2012). Discoloration of the crown is a frequent observation, and continued growth of the root is not predictably achieved (Kahler et al., 2014; Simon et al., 2014).

The procedure of “controlled bleeding” *per se* is problematic from a clinical point of view, mainly for two reasons: Hemorrhage has a negative effect on tooth color (Marin et al., 1997), and the whole procedure and its state-of-the-art execution are hard if not impossible to be monitored. Proper case documentation, however, is the core of good clinical practice. Recent approaches to replace the controlled bleeding step in regenerative endodontics involved the application of a fibrin hydrogel into the empty pulp space in order to create a synthetic, non-staining blood clot (Ruangsawasdi et al., 2014). The fibrin hydrogel could be placed in a controlled manner using a micro-cannula or a lentulo spiral. However, it would be desirable to obtain a radiopaque hydrogel for better controlling and monitoring of the procedure. In the current study we investigated the effects of a biocompatible iodinated X-ray contrast agent systemically used in angiography on some basic properties of a fibrin hydrogel intended for endodontic applications.

## Materials and methods

### Preparation of hydrogels

Hydrogels were prepared from a commercially available fibrin sealant (Tisseel, Baxter; Deerfield, IL). The two components of the sealant were diluted to obtain a control fibrin hydrogel as previously described (Ruangsawasdi et al., 2017). To prepare the control hydrogels, the thrombin component and the fibrinogen component were diluted in Tris-buffered saline (TBS) and then mixed as described below. In the test hydrogels, the TBS was replaced by an aqueous isotonic contrast agent (Visipaque 320, GE Healthcare, Cork, Ireland) containing 652 mg of iodixanol per mL of liquid, which equals 320 mg/mL of iodine. The pH of the two solutions was measured using a calibrated electrode (Metrohm, Herisau, Switzerland). The pH of TBS was 7.6, while the pH of Visipaque 320 equaled 7.1.

### Gelation kinetics

Fibrin hydrogels gelate via fibrin clotting. Clotting time was tested according to the method described by Vermylen et al. (1963). The two hydrogel components were prepared separately in micro-centrifugation tubes in a water bath at 37°C. For 250 μL of the thrombin solution 2 μL of thrombin were added to 248 μL of TBS or Visipaque; 250 μL of fibrinogen solution were prepared by adding 44 μL of fibrinogen to 206 μL of TBS or Visipaque. Pre-warmed thrombin solution was transferred to the fibrinogen counterpart using this pipette. The agents were mixed using a pipette. The micro-centrifugation tubes were kept in the water bath at 37°C during the whole experiment. A platinum loop was moved in and out of the clotting mixture until the appearance of the fibrin web, which marked the end point (hydrogelation). The time to reach this point was recorded using a stopwatch. These experiments were performed in triplicates with freshly prepared solutions.

### Assessment of radiopacity

**Figure 1:**
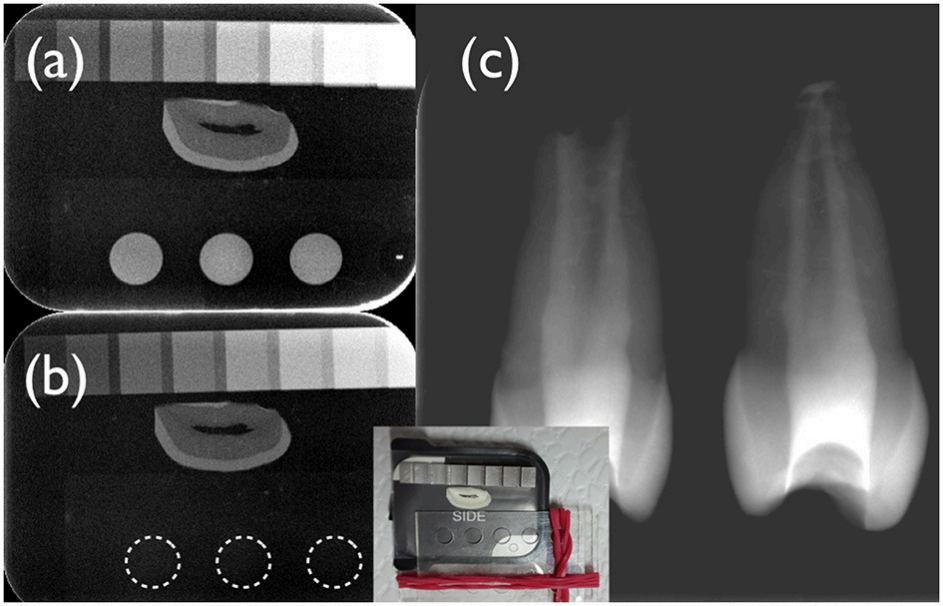
**Digital radiographs of the test (a,c)** and the control fibrin hydrogel **(b)** under investigation. A cross-section of a human incisor crown was used as a reference. A photograph of the set-up is depicted in the insert. The aluminum step-wedge had a thickness of 0.5, 1, 1.5, 2, and then 3 mm Al, followed by 1 mm steps. As can be appreciated from this figure, the test hydrogel **(a)** had a radiopacity of ~2 mm Al/mm, whilst the control hydrogel **(b)** displayed no radiopacity (dotted lines). Freshly mixed test hydrogel containing iodixanol was placed in the root canal system of immature human premolars using a lentulo spiral **(c)**. This test hydrogel apparently filled the whole canal space and could be clearly discerned from root dentin.

Test and control hydrogels were filled into polycarbonate molds 10 mm in diameter and 1 mm thick. Three molds were filled per experiment. Slices with a thickness of 1 mm from the crowns of bovine teeth served as control for the clear distinction between material and the surrounding tissue in clinical situations. Specimens were placed on a radiographic sensor (Digora, Soredex, Tuusula, Finland) together with an aluminum step-wedge with a thickness from 0.5 to 6 mm (0.5 and 1 mm increments, Figures 1a,b). A Trophy Irix (Trophy, Paris, France) X-ray unit operating at 65 kV, 8 mA, and 0.22 s with a focus-film distance of 25 cm was used. Hydrogels were prepared immediately prior to use. These triplicate measurements were performed three times each with freshly prepared hydrogels. Digital radiographic images were imported with the Digora system, using Digora software version 1.51 for Windows without gray scale correction. Optimas image analysis software (Meyer Instruments Inc., Houston, TX) was used to determine the gray value of the samples and convert these to mm of aluminum equivalent (mm Al). To assess the relative radiopacity of the test fibrin hydrogel in human teeth, the root canals of premolars extracted for orthodontic reasons were prepared using the ProTaper system (Sirona Dentsply Endodontics, Ballaigues, Switzerland) and filled with freshly mixed hydrogel using a lentulo spiral. The teeth were radiographed as described above.

### Swelling of hydrogels

The water-absorbing capacity (swelling) was calculated gravimetrically according to the formula:

$$\text{Swelling ratio}=(w_{s}-w_{d})/w_{d}$$

where *w*_*s*_ is the weight of the swollen hydrogel and *w*_*d*_ is the weight of the dry hydrogel. The hydrogels were prepared with TBS or Visipaque, frozen at −80°C overnight and freeze-dried under vacuum for 24 h until constant weight in a lyophilization apparatus (Christ, Osterode am Harz, Germany). The dry hydrogels were pre-weighed in a precision balance (PE360; Mettler Toledo, Greifensee, Switzerland) and were immersed in 5 mL of TBS at 37°C for 5 min, 1, 8, and 24 h. After removing them from the TBS at each time point, the hydrogels were gently wiped with a filter paper and weighted again. Subsequently, the hydrogels were immersed in fresh TBS. The swelling ratio was measured by comparing the weight of hydrogels before and after immersing in TBS according to the equation. These experiments were performed in triplicates.

### Scanning electron microscopy

To visualize the microstructure of the test and the control hydrogels under investigation, triplicates of lyophilized specimens were inspected using scanning electron microscopy (SEM). To this end, the freeze-dried hydrogels were attached to sticky carbon pads (Plano, Wetzlar, Germany) on SEM pin stubs. Specimens were sputtered with an 8 nm gold layer (Safematic, Bad Ragaz, Switzerland). Images were obtained at 5 mV acceleration voltage and 12 mm working distance using a secondary electron detector (Zeiss Supra 50VP, Oberkochen, Germany).

### Release of iodixanol from test hydrogels

The UV spectrum of an iodixanol solution in water shows a maximum at 244 nm and a specific absorption coefficient of 320 l g^−1^ cm^−1^ at that wavelength (Schroder et al., 1997). To test the release of iodixanol from the test hydrogels, 1 mL of hydrogel was mixed with 49 mL of TBS in 50 mL polypropylene centrifugation tubes (Falcon, Thermo Fisher Scientific, Waltham, MA). These tubes were rotated at room temperature (25°C) at 6 rpm in an overhead rotator with a radius of 10 cm (Bibby Scientific, Staffordshire, UK). 100 μL of the solution were removed (and not replenished) to be assessed spectrophotometrically for their iodixanol content after 30 min, 1, 3, 8, 24, 48, 72, 120, and finally after 168 h. Experiments were done in triplicates. Iodixanol that washed out from the hydrogel was quantified at 244 nm in a spectrophotometer (Shimadzu, Kyoto, Japan) in quartz cuvettes against a standard curve. Iodixanol wash out is presented as % of total iodixanol originally present in the hydrogel in solution.

### Chick chorioallantoic membrane (CAM) assay

CAM assays were performed to assess the impact of test and control hydrogels on tissue viability and vascularization of fertilized chicken eggs (Beckers et al., 1997). A total of 14 fertilized Lohman white LSL chicken eggs (Animalco AG, Staufen AG, Switzerland) were used for these experiments, seven in the control and seven in the test hydrogel group. According to the local animal care guidelines (Canton of Zurich, Switzerland), no approval was necessary to perform these experiments, as they were executed only until embryonic day (ED) 14. Detailed procedures have been published (Woloszyk et al., 2016). Eggs were pre-incubated for 3 days at 38°C at a rotational speed of 360°/4 h (Bruja 3,000, Brutmaschinen-Janeschitz GmbH, Hammelburg, Germany). On ED 3, the eggs were processed for *in ovo* cultivation. The eggshell was wiped with 70% ethanol. To lower the developing embryo before opening of the shell using Scotch tape and scissors, 4 mL of albumen were removed through a small hole in the shell. The egg was stabilized in a 60 mm Petri dish base. The opening was covered with a second 60 mm Petri dish base, which was fixed to the bottom base using Scotch tape. Subsequently, the eggs were incubated at 37°C. On ED 7, hydrogel samples polymerized within a silicone O-ring (from Corning cryogenic vials, Sigma-Aldrich, St. Louis, MO) were gently placed on the CAM (1 per egg), and the eggs were incubated for further 7 days until ED 14. One egg in the test and one in the control group did not survive the 7 days pre-incubation, leaving 6 viable eggs in the control and the test group, respectively. On ED 14, the hydrogel probes together with the CAM were cut out and after fixing in 4% paraformaldehyde for 60 min at room temperature, the probes were washed and stored in PBS. Pictures were taken using a stereomicroscope (Leica Microsystems AG, Heerbrugg SG, Switzerland). Vascularization within the silicone O-ring was quantified on these images using ImageJ v1.50b.

### Data presentation

Numerical data is presented as means and standard deviations. Pair-wise comparisons between the test and the control hydrogel were performed using paired *t*-test. The alpha-type error was set at 5% (*p* < 0.05).

## Results

The control fibrin hydrogel prepared with TBS clotted quickly. The recorded mean gelation time was 22 ± 1 s. Gelation was strongly delayed by the presence of iodixanol; it occurred after 1,580 ± 75 s (*p* < 0.05), i.e., 26 min. The iodixanol solution (Visipaque) rendered the test hydrogel radiopaque with a mean radiopacity of 1.9 ± 0.2 mm Al/mm. The test hydrogel could clearly be distinguished from root dentin of human teeth radiographically (Figures 1a,c), while the control hydrogel had no detectable radiopacity (Figure 1b).

**Figure 2:**
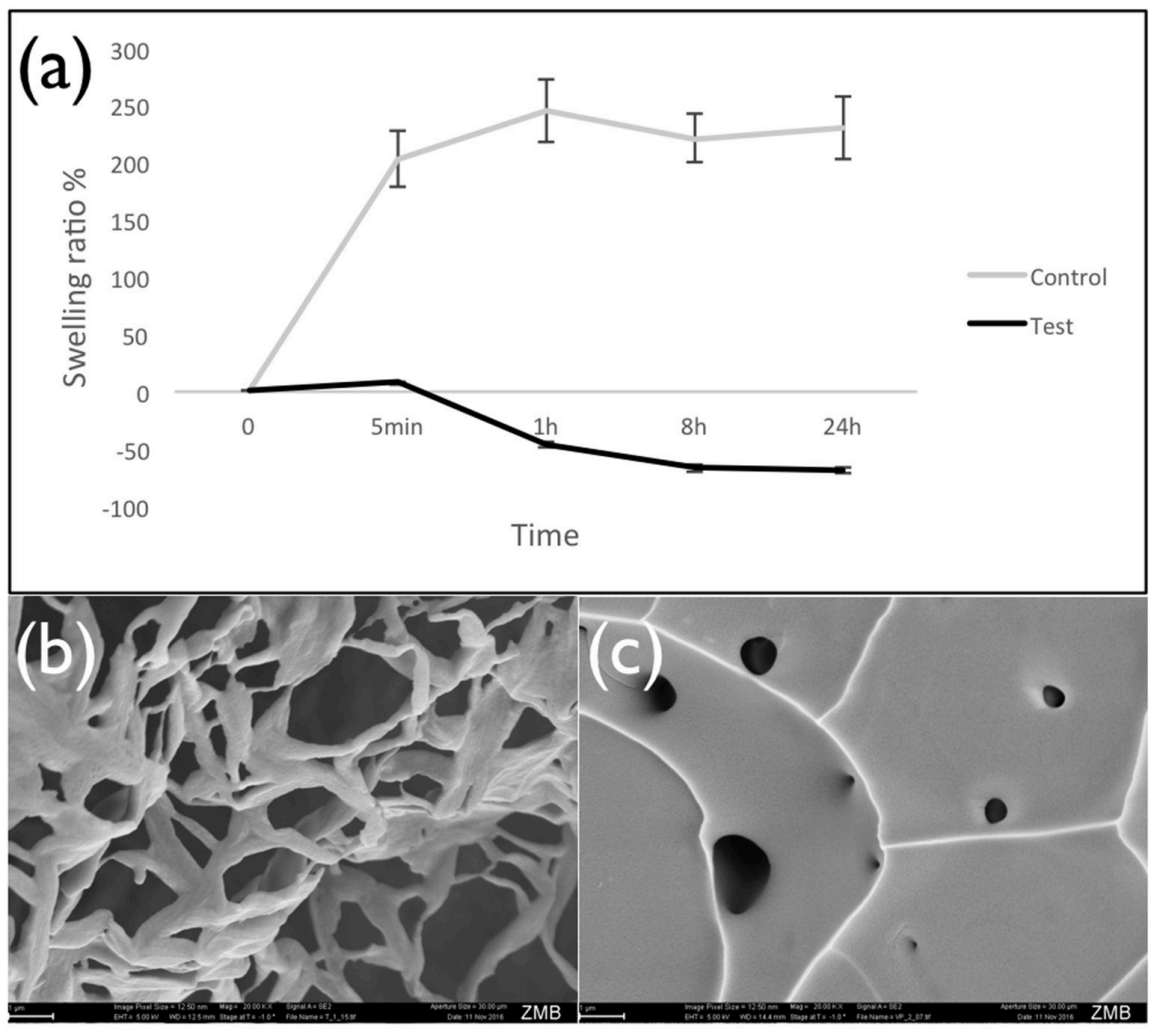
**Swelling of lyophilized hydrogels**. Panel **(a)** presents the swelling ratio of lyophilized fibrin hydrogels prepared with TBS (control) and the iodixanol solution (Visipaque) after different time of incubation in TBS at 37°C, showing that iodixanol reduced the water sorption of hydrogel. As time passed, the amount of retained water in the test hydrogel decreased and caused the shrinking of the hydrogel structure. Panel **(b)** is a typical SEM image of the control hydrogel, panel **(c)** a corresponding image of the test hydrogel containing iodixanol.

Results showed that swelling was strongly reduced when iodixanol was added to the hydrogel. Values decreased from 203% for control hydrogels prepared with TBS to only 8% for counterparts prepared with Visipaque (time point 5 min, *p* < 0.05) (Figure 2a). Inspection of the lyophilized hydrogels in a scanning electron microscope confirmed that iodixanol influenced their structure. While the control hydrogel showed the typical sponge-like porous structure (Figure 2b), the test hydrogel containing iodixanol had a more homogenous appearance with few internal openings (Figure 2c).

**Figure 3:**
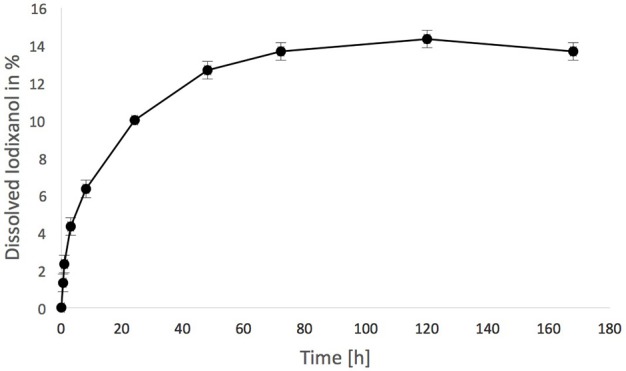
**Iodixanol release from test fibrin hydrogels**. One milliliter was suspended in 49 mL of tris-buffered saline (TBS) over time, expressed in % of the total iodixanol that was present in the hydrogel initially.

**Figure 4:**
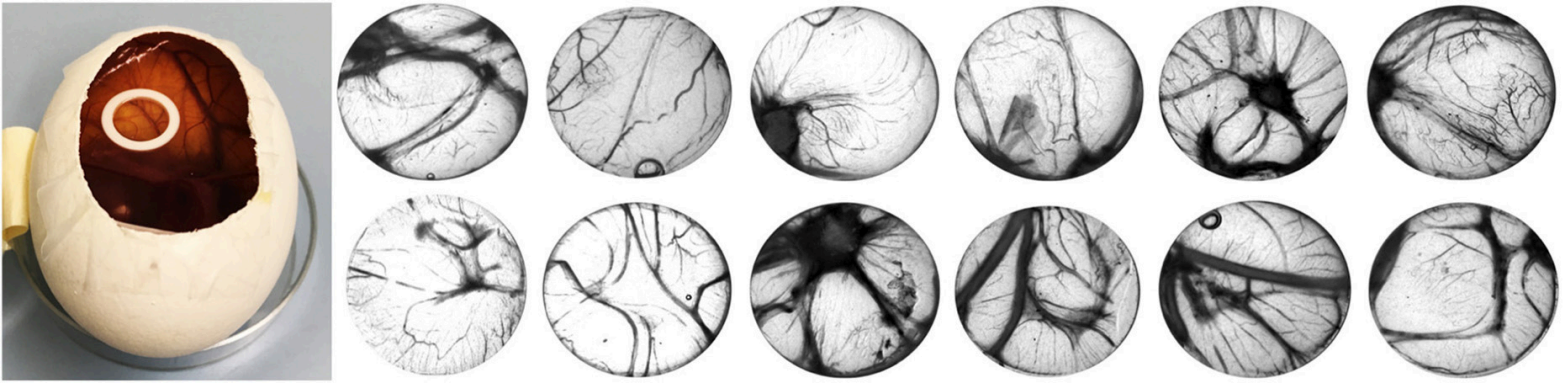
**Chick chorioallantoic membrane (CAM) assay used in this study (left)**. Control fibrin hydrogels (upper row) and radiopaque test counterparts spiked with iodixanol (lower row) were placed in o-rings on the CAM for 7 days (from embryonic day 7 to 14), and vascularization was quantified. There was no difference between the test and the control group.

Iodixanol release from the hydrogels prepared with Visipaque reached 14.5 ± 0.5% after 120 h and then ceased (Figure 3). This release from test hydrogels did not have any apparent effect on the fertilized chicken eggs. All incubated eggs survived the exposure to either the test or the control hydrogel between embryonic day 7 and 14, and vascularization in the chorioallantoic membrane under the hydrogels looked similar (Figure 4). Quantification of the vascularization revealed no statistically significant difference between groups (*p* = 0.34).

## Discussion

This study showed that a radiopaque and tissue-compatible, yet slow setting and less porous fibrin hydrogel is obtained by diluting a commercially available fibrin sealant with an isotonic contrast agent containing iodixanol rather than TBS. Basic properties of this experimental hydrogel such as hydrogelation kinetics, radiopacity, swelling, iodixanol release, and overall tissue compatibility were assessed. The current *in vitro* and *in ovo* results are preliminary observations and should be seen as such. At this point, it is not possible to draw conclusions regarding the suitability of the test hydrogel for pulp tissue engineering.

The fibrin sealant used in this study (Tisseel), which is designed to achieve hemostasis, to seal or glue tissue, and to support wound healing, has to be diluted in TBS to obtain a fibrin hydrogel that could be invaded by cells during the pulp regeneration process (Ruangsawasdi et al., 2014). This creates an opportunity to replace the TBS used for that purpose with a similar, isotonic product having enhanced contrast properties (Visipaque 320). Opacity of hydrogels by addition of the iodixanol that is contained in Visipaque has recently been tested in chitosan hydrogels for controlled blood vessel embolization (Fatimi et al., 2016). Iodixanol extends blood-clotting time (Bellemain-Appaix et al., 2012), which is concurrent with the results obtained here using a fibrin sealant. It is a non-ionic dimer (Jones and Goodall, 2003). In the current approach, we chose iodixanol because alternative contrast agents such as the non-ionic monomer iohexol (Omnipaque, Bayer Pharma AG, Berlin, Germany) and the ionic dimer ioxaglate (Hexabrix, Guerbet, Roissy, France) appear to have an even higher anticoagulant effect than iodixanol (Corot et al., 1996). It has also been shown that the slowing of the hydrogelation process in chitosan hydrogels by iodixanol is dose-dependent (Fatimi et al., 2016). Since teeth are inherently radiopaque structures, we used a high concentration of iodixanol compared to previous experiments on chitosan hydrogels for embolization. However, the radiopacity of permanent sealing materials requested by the ISO norm (2012), which is 3 mm Al/mm, was still not met. Nevertheless, the preliminary filling experiments with human teeth showed that the radiopacity should be sufficient for the intended purpose, i.e., the monitoring of the correct placement of the fibrin hydrogel (Figure 1c).

The extended clotting time, which resulted from the presence of iodixanol in the fibrin hydrogel, can be desirable in endodontic applications. The radiopaque fibrin hydrogel under investigation has a working time similar to endodontic sealers (Whitworth, 1999). It can be applied into the root canal system using for example a lentulo spiral. In contrast, the mere fibrin hydrogel diluted with TBS, which is commonly used as a control (Ruangsawasdi et al., 2014, 2017), is not easy to administer into the root canal system because of its immediate gelation.

The swelling of hydrogels is related to the crosslinking ratio in their polymeric network. Highly cross-linked hydrogels tend to swell less than counterparts with a lower crosslinking ratio (Peppas et al., 2000). The effect of iodixanol on swelling of lyophilized hydrogels and on the apparent hydrogel structure in SEM scans strongly suggests that hydrogel porosity is reduced. This is in line with previous studies showing reduction of chitosan hydrogel swelling by increasing their iodixanol concentration (Fatimi et al., 2016). The addition of iodixanol to fibrin-rich clots resulted in shorter, thinner, and more numerous fibrin fibers when compared to counterparts made with ioxaglate or buffer, leading to a much more compact 3D architecture with smaller pores and higher crosslinking of fibrin fibers (Bellemain-Appaix et al., 2012). Furthermore, there was an apparent negative swelling after 5 min with the test hydrogel under the current conditions. This might be indicative of a material degradation process. The iodixanol modified hydrogels hardly took up any TBS fluid, suggesting an important loss of hydrophilicty or material permeability. Both are important characteristics for a biomaterial scaffold in regenerative procedures. Incorporation of iodixanol seems to produce a fiber coalescence process leading to a tensional film-like morphology (Figure 2). It thus remains to be investigated how these iodixanol-induced alterations influence the permeability of fibrin hydrogels to migrating cells during regenerative processes.

We used the CAM assay as a measure of tissue compatibility of the new radiopaque hydrogel under investigation. Although this assay is relatively simple, it can be considered suitable for the pre-screening of scaffolds to determine whether they cause any adverse tissue reactions (Baiguera et al., 2012). Our results suggest good tissue compatibility of the test as well as the control hydrogel. However, it needs to be cautioned that the CAM assay is a non-mammalian organism that has not yet developed an immune system. This assay merely determined the effect of the fibrin hydrogels on tissue viability and vascularization. Histological assessment of the hydrogels revealed that no blood vessels grew into the body of the hydrogels (data not shown). This is to be expected, as we did not embed any chemotactic molecules into the hydrogels (Anderson et al., 2011).

In conclusion, the current results revealed the possibility to elaborate radiopaque fibrin hydrogels for potential endodontic applications from materials that are already commercially available. Under the current conditions, tissue compatibility appeared not to be influenced by the addition of iodixanol to the hydrogel. Further *in vivo* studies should test the effects of different contrast agents on fibrin hydrogel structure combined with cell migration and attraction.

## Author contributions

Study design: GH, MZ, TM, and FW. Data collection: GH, AW, and AI. Data analysis: MZ. Drafting manuscript: GH and MZ. Revising manuscript content: GH, MZ, AW, TM, AI, and FW. Approving final version of manuscript: GH, MZ, AW, TM, AI, and FW. MZ takes responsibility for the integrity of the data analysis.

## Funding

This research was partly supported by the Swiss National Foundation (SNSF) grant 31003A_135633 (TM and AW), and a Swiss government scholarship (AI).

### Conflict of interest statement

The authors declare that the research was conducted in the absence of any commercial or financial relationships that could be construed as a potential conflict of interest.
